# Mutational status of synchronous and metachronous tumor samples in patients with metastatic non-small-cell lung cancer

**DOI:** 10.1186/s12885-016-2249-6

**Published:** 2016-03-11

**Authors:** Gilles Quéré, Renaud Descourt, Gilles Robinet, Sandrine Autret, Odile Raguenes, Brigitte Fercot, Pierre Alemany, Arnaud Uguen, Claude Férec, Isabelle Quintin-Roué, Gérald Le Gac

**Affiliations:** CHRU de Brest, Institut de Cancérologie et d’Hématologie, Brest, France; CHRU de Brest, Hôpital Morvan, Bat 5 bis, Laboratoire de Génétique Moléculaire et d’Histocompatibilité, 2 Avenue Foch, 29200 Brest, France; Plateforme de Génétique Moléculaire des Cancers (INCa), Brest, France; Inserm U1078, Université de Brest, SFR SnInBioS, Brest, France; CHRU de Brest, Service d’Anatomopathologie, Brest, France

**Keywords:** Non-small-cell lung cancer, Metastatic lesion, Rebiopsy, Genetic biomarkers, Targeted therapy

## Abstract

**Backgrounds:**

Despite reported discordance between the mutational status of primary lung cancers and their metastases, metastatic sites are rarely biopsied and targeted therapy is guided by genetic biomarkers detected in the primary tumor. This situation is mostly explained by the apparent stability of *EGFR*-activating mutations. Given the dramatic increase in the range of candidate drugs and high rates of drug resistance, rebiopsy or liquid biopsy may become widespread. The purpose of this study was to test genetic biomarkers used in clinical practice (*EGFR*, *ALK*) and candidate biomarkers identified by the French National Cancer Institute (*KRAS*, *BRAF*, *PIK3CA*, *HER2*) in patients with metastatic non-small-cell lung cancer for whom two tumor samples were available.

**Methods:**

A retrospective study identified 88 tumor samples collected synchronously or metachronously, from the same or two different sites, in 44 patients. Mutation analysis used SNaPshot (*EGFR*, *KRAS*, *BRAF* missense mutations), pyrosequencing (*EGFR* and *PIK3CA* missense mutations), sizing assays (*EGFR* and *HER2* indels) and IHC and/or FISH (ALK rearrangements).

**Results:**

About half the patients (52 %) harbored at least one mutation. Five patients had an activating mutation of *EGFR* in both the primary tumor and the metastasis. The T790M resistance mutation was detected in metastases in 3 patients with acquired resistance to *EGFR* tyrosine kinase inhibitors. FISH showed discordance in *ALK* status between a small biopsy sample and the surgical specimen. *KRAS* mutations were observed in 36 % of samples, six patients (14 %) having discordant genotypes; all discordances concerned sampling from different sites. Two patients (5 %) showed *PI3KCA* mutations. One metastasis harbored both *PI3KCA* and *KRAS* mutations, while the synchronously sampled primary tumor was mutation free. No mutations were detected in *BRAF* and *HER2*.

**Conclusions:**

This study highlighted noteworthy intra-individual discordance in *KRAS* mutational status, whereas *EGFR* status was stable. Intratumoral heterogeneity for *ALK* rearrangement suggests a limitation of single-biopsy analysis for therapeutic strategy with crizotinib.

**Electronic supplementary material:**

The online version of this article (doi:10.1186/s12885-016-2249-6) contains supplementary material, which is available to authorized users.

## Background

Chemotherapy is still the standard treatment for metastatic non-small-cell lung cancer (NSCLC), which represents 60 % of cases at diagnosis. Overall and progression-free survival rates have gradually improved, notably with the use of different drugs or combinations and more precise histological classification [1,2]. However, median survival in advanced disease is still less than 12 months, even among patients receiving platinum-based doublet chemotherapy and bevacizumab [1,3]. The recent introduction of tyrosine kinase receptor inhibitors for the *EGFR* and *ALK* genes [4–8], which are mutated in respectively 10 and 4 % of non-small-cell lung tumors in Caucasian patients, has had a major impact, despite occasional resistance mutations such as T790M in the *EGFR* gene, which is found in more than 50 % of patients treated by tyrosine kinase inhibitor (TKI) [9,10]. Several clinical trials are underway, based on genetic biomarkers and activation pathway inhibitors. The intracellular oncogene *KRAS* is a particularly attractive target because of its high mutation rate (>25 % of patients), especially in current and former heavy smokers [11].

A major issue raised by targeted therapies is potential discordance between the mutational status of the primary tumor and its metastases, or between two regions of the same tumor. This is particularly important in lung cancer: repeat biopsy is rarely performed [12], even though various studies have shown discrepancies in *EGFR, ALK* and *KRAS* mutational status [13–18].

The present study examined discordance between repeat samples from the same tumor site or samples from two different sites, collected synchronously or metachronously. The principal mutations of *EGFR, ALK, KRAS, BRAF, PIK3CA* and *HER2* were analyzed in 44 patients with non-small-cell lung cancer. The *KRAS*, *BRAF*, *PIK3CA* and *HER2* oncogenes were selected because they represented potential drug targets [19]. They were identified as potentially predictive biomarkers in NSCLC by the French National Cancer Institute (INCa) and were introduced in the French nationwide initiative for tumor molecular profiling during the 2010–2014 period [20].

## Methods

### Patients

This retrospective cohort study included patients with non-small-cell lung cancer (adenocarcinoma or squamous cell carcinoma) for whom two tumor samples were available, collected synchronously or metachronously either from the same site or from two different sites during disease course between 2005 and 2012. Patients were identified by cross-matching information from surgical files (surgical biopsy of metastasis, analysis of lobectomy or pneumonectomy specimen, or bronchial biopsy) with the medical codes of the institution. The corresponding tissue blocks were identified in each case. Samples were obtained by simple biopsy (*n* = 47; 53.4 %), surgical excision or biopsy (*n* = 39; 44.3 %), or fine-needle aspiration cytology (*n* = 2; 2.3 %).

### Ethics statement

The study was conducted in accordance to the Declaration of Helsinki principles. It was approved by the Human Research Ethics Committee of Brest University Hospital (“*Comité de Protection des Personnes* - Ouest VI”; January 18, 2012). Written informed consent for the use of tissues and clinical data for research was taken from patients at the time of procurement of tumor specimens.

### DNA extraction

All tumor samples were formalin-fixed and embedded in paraffin (FFPE). In each case, the percentage of tumor cells was determined by an experienced pathologist on a representative histological cross-section. Samples from at least three serial 10-μm sections were macrodissected and pooled for DNA extraction. DNA was extracted using the Maxwell® 16 FFPE Plus LEV DNA purification kit (Promega, Madison, WI, USA) according to the manufacturer’s instructions.

### Mutational analyses

#### EGFR, KRAS, BRAF and PI3KCA status

Fragment-length analysis was used to screen for deletions and insertions in *EGFR* exons 19 and 20 and in *HER2* exon 20. Genomic tumor DNA was amplified using the Qiagen™ Multiplex PCR kit (Qiagen, Hilden, Germany) with the following primers: 5′-N-CTG-GAT-CCC-AGA-AGG-TGA-GA-3′ and 5′-GAT-TTC-CTT-GTT-GGC-TTT-CG-3′ (*EGFR* exon 19), 5′-N-CTC-CAG-GAA-GCC-T AC-GTG-AT-3′and 5′-CTG-CGT-GAT-GAG-CTG-CAC-3′ (*EGFR* exon 20), and 5′-N-CCT-CTC-AGC-GTA-CCC-TTG-TC-3′ and 5′-AGG-GCA-TAA-GCT-GTG-TCA-CC-3′ (*HER2* exon 20). For universal labeling, the forward primers were tailed with a short nucleotide sequence (N) that matched a universal FAM-labeled probe [21]. The labeled PCR products were subjected to capillary electrophoresis on an ABI PRISM 3100 XL genetic analyzer (Applied Biosystems, Courtabœuf, France) and compared with the wild-type PCR product to determine whether differences in length were present and represented deletion or insertion. Positive samples were re-amplified and sequenced using the BigDye Terminator v.1 cycle sequencing kit (Applied Biosystems), according to the manufacturer’s protocol. Sequence electrophoregrams were interpreted using SeqPatient analysis software version 3.5.2 (JSI Medical Systems, Ettenheim, Germany).

The *EGFR, KRAS* and *BRAF* genes were analyzed for presence of missense mutations using the ABI PRISM SNaPshot Multiplex kit (Applied Biosystems). Briefly, three multiplex PCRs were designed, the first for *KRAS* exon 2 (codons 12 and 13) and *BRAF* exon 15 (codon 600), the second for *KRAS* exon 3 (codon 61) and 4 (codon 146) and the third for *EGFR* exons 18 (codon 719) and 20 (codon 790). Multiplex PCR used the Qiagen™ Multiplex PCR kit with a total volume of 20 μL. PCR products were treated with Exonuclease I (ExoI) and shrimp alkaline phosphatase (SAP) (USB, Cleveland, Ohio, USA). Each extension primer (SNaPshot primer) was designed to anneal to the reverse strand of its targeted PCR product adjacent to the mutation site of interest. SNaPshot primers contained an additional tail at their 5’ end for simultaneous detection. Mutation detection reactions were performed in a total volume of 5 μL, comprising 1.5 μL SAP/ExoI-treated PCR product, 2 μL SNaPshot Multiplex Ready Reaction mix and 1.5 μL SNaPshot primer mix (each primer at a final concentration of 0.5 to 1.5 μM). Products were treated with SAP before automated sequencing (ABI PRISM 3500 Dx Genetic Analyzer, Applied Biosystems). Data were analyzed with GeneMapper Analysis software version 4.0 (Applied Biosystems).

The *EGFR* L848R mutation and *PIK3CA* exons 10 and 21 (codons 542, 545, 546, 1043, 1044, 1047 and 1049) were analyzed by pyrosequencing. For each target, PCR amplification was performed using the Qiagen™ Multiplex PCR kit with one of the primers biotinylated. Biotinylated products were immobilized on streptavidin-coated beads. After washing steps, DNA samples were released by denaturation in NaOH and annealed into single strands to a sequencing primer. Pyrosequencing was performed on a PyroMark Q24 system (Qiagen) following the manufacturer’s instructions. PCR primers, sequencing primers and dispensing orders are available upon request.

#### ALK status

*ALK* rearrangement was investigated in FFPE samples (3 μm thick) by Fluorescent In Situ Hybridization (FISH) and/or ImmunoHistoChemistry (IHC). FISH was performed on Superfrost ® Plus slides (Thermo Scientific, Saint-Herblain, France) with the Vysis LSI ALK Dual Color break-apart rearrangement probe (Abbott Molecular, Abbott Park, IL, USA). The slides were read using a Carl Zeiss epifluorescence microscope and the ISIS digital image analysis system (Isis in situ imaging system v.5.3, Metasystems, Altlussheim, Germany). FISH-positive cases were defined as those presenting >15 % split signals or an isolated orange signal in tumor cells. At least 100 nuclei were assessed for each tumor sample. Immunostaining with ALK monoclonal antibody (1:25, clone 5A4, Clinisciences, Nanterre, France) was performed using the Ventana Benchmark XT® automated slide preparation system and the OptiView DAB IHC Detection Kit according to the manufacturer’s instructions (Roche Diagnostics, Mannheim, Germany). ALK IHC scores, based on staining intensity and the percentage of tumor cells with positive cytoplasmic staining, were assigned as follows: 0, no stained cells; 1, faint or weak staining in >5 % cells or any staining intensity in ≤5 % of tumor cells; 2, moderate staining intensity in >5 % of tumor cells; or 3, strong granular staining intensity in >5 % of tumor cells.

### Availability of data and materials

A dataset (mutations detection) supporting the conclusions of this article is available in Additional file 1: Figure S1.

### Statistical analyses

Fisher’s exact test was used to identify significant factors for discordance in mutational status between two samples from a given patient.

## Results

### Study population

Table 1 summarizes demographic characteristics. There were 44 patients (27 male, 17 female) for whom two samples could be analyzed by molecular biology (primary tumor and metastasis or two samples from the same site). Current or former heavy smoking (>10 pack years) was reported in the majority of cases (32/44). Mean age at diagnosis was 60.5 years, slightly below the French national average [22]. More than half the patients had stage IV disease at diagnosis. All patients had non-small-cell lung cancer; the predominant histological type was adenocarcinoma (41/44). Synchronous samples were defined as being obtained within a 3-month period during which no cancer treatment was administered.

**Table 1:** Patient characteristics

	Number	Total (%)
Age (years)	60.5 [39–76]	
gender		
men	27	61.3
women	17	38.7
Never smokers	7	16
Current and former smokers	32	72.7
unknown	5	
Histology		
Adenocarcinoma	41	93
Squamous cell	3	7
Stage at diagnosis		
I	6	13.7
II	4	9.1
III	9	20.4
IV	25	56.8
Average time between samples (months)		
Synchronous	1.2 (*n* = 13)	
métachronous	18 (*n* = 31)	

**Fig. 1:**
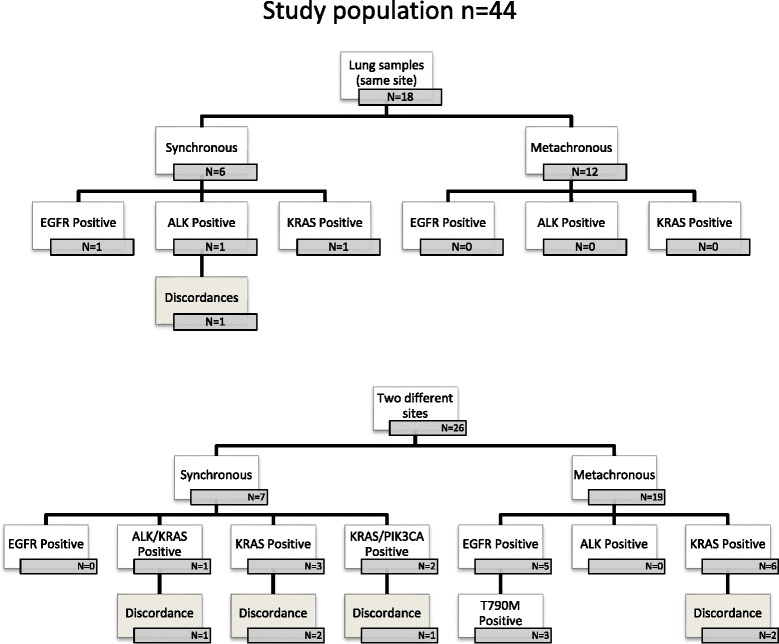
Distribution of multigene mutations and discordances (*EGFR*, *KRAS*, *BRAF*, *HER2* and *PIK3CA*) in 44 French patients with metastatic non-small-cell lung cancer. The study population was composed of 26 patients sampled at different sites and 18 patients sampled twice at the same primary lung cancer location. Synchronous or metachronous status was taken into account in each group

The population was divided into patients sampled twice at the same pulmonary site (18 patients: 12 synchronous and six metachronous samples), and patients sampled at two different sites: lung and another organ, two different pulmonary sites, or two organs other than the lung (26 patients: six synchronous and 20 metachronous samples) (Fig. 1). Remote metastases were situated in the brain (*n* = 8), lung (*n* = 5), pleura (*n* = 4), bone (*n* = 3), lymph node (*n* = 4), liver (*n* = 3) or skin (*n* = 2).

### Discordance in mutational status

About half the patients (52 %) harbored at least one mutation. The distribution of the mutations detected in the 44 patients and 88 samples is shown in Fig. 1.

#### EGFR, KRAS, BRAF and PI3KCA mutations

**Fig. 2:**
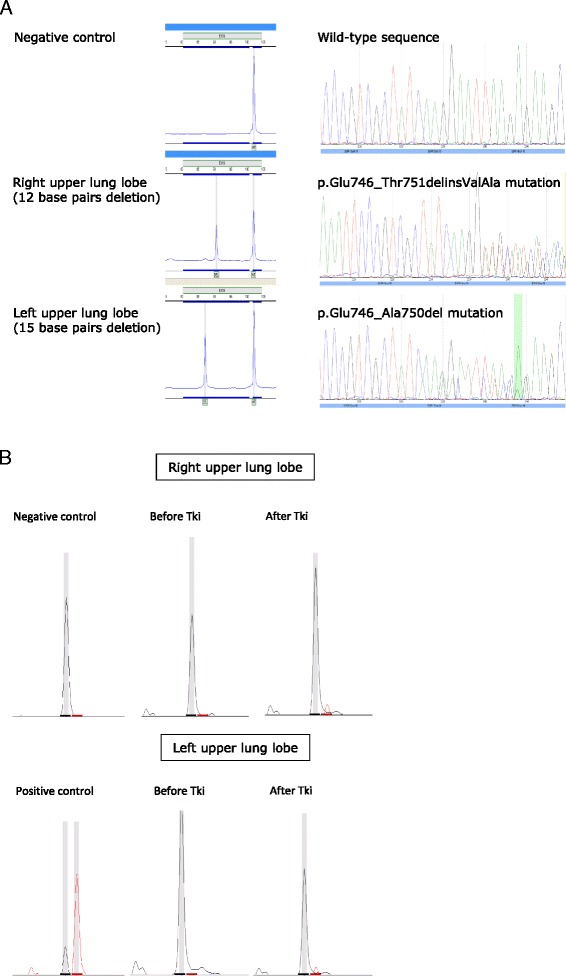
Screening for *EGFR* exon 19 deletions and the T790M resistance mutation in two independent tumors (*right* versus *left* upper lung lobe) diagnosed synchronously. **a** Fragment-length analysis and Sanger sequencing demonstrating two different *EGFR* deletions in the two independent tumors. **b** SNaPshot analysis showing simultaneous emergence of the T790M resistance mutation in the two tumors after completion of EGFR TKI therapy

Five patients had an activating mutation of *EGFR* (L858R, or exon 19 deletion) in both the primary tumor and the metastasis. No discordances were found in these cases. None of these patients had additional mutations in *KRAS, BRAF* or *PI3KCA*. One discordance was not taken into account for analysis, as the patient, a non-smoking female, appeared to have two synchronous tumors; both these primary tumors were tested for *EGFR* activating mutations. As shown in Fig. 2, the tumor in the right upper lobe harbored an apparent 12 bp deletion corresponding to the p.Glu746_Thr751delinsValAla mutation (COSMIC mutation ID: COSM53205), while the tumor in the left upper lobe harbored a 15 bp deletion resulting in the p.Glu746_Ala750del mutation (COSM6225). These two distinct *EGFR* mutations were confirmed on repeat biopsy several months later. It is noteworthy that both tumors progressed synchronously under TKI treatment, and were subsequently found to be p.Thr790Met (T790M)-positive (COSM6240).

In all, the p.Thr790Met (T790M) resistance mutation was found in metastases in three patients, but this was not considered as discordance because the initial activating mutation was still present in the metastasis.

Six cases of discordance in *KRAS* status were found: 3 between synchronous samples and 3 between metachronous samples. Three of the discordances involved *KRAS* mutation in the metastasis, while mutations were detected only in the primary tumor in another patient. The remaining two patients harbored different mutations in the primary tumor and in the metastasis. Interestingly, the G12C mutation (COSM516) was detected in eight of the 12 patients with *KRAS* mutation in the primary tumor and/or metastasis. All these patients were current heavy smokers.

Two patients showed *PI3KCA* mutations: p.His1047Arg (H1047R; COSM775) and p.Glu542Lys (E542K; COSM760). It is noteworthy that *PI3KCA* E542K mutation was concomitant with *KRAS* G12C mutation in the metastasis of a man in whom the synchronously sampled primary tumor was mutation-free.

#### ALK gene rearrangements

**Fig. 3:**
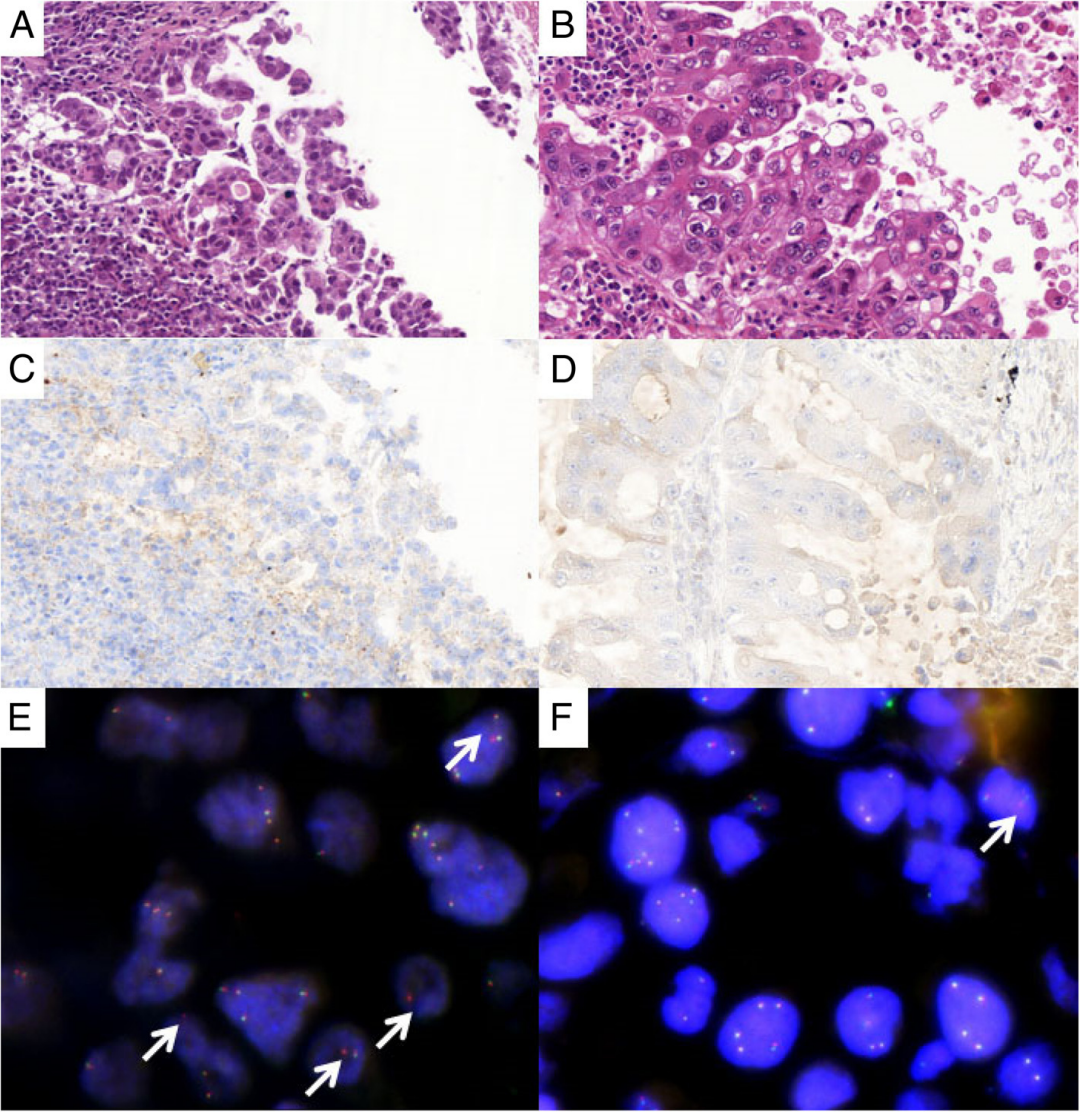
*ALK* rearrangement status and ALK expression determined by fluorescence in-situ hybridization (FISH) and immunohistochemistry (IHC) in two regions of the same primary lung tumor. **a**, **b** examination after hematoxylin-eosin-safran (HES) staining (×400): histological features of adenocarcinoma from the bronchoscopy (**a**) and the surgical resection (**b**) specimens. **c**, **d** ALK expression: very faint positive immunoreactivity (score, 0/1) in both the small biopsy and the corresponding surgical resection specimen. **e**, **f** Typical break-apart pattern observed by FISH (*arrow*): 25 % of rearranged tumor nuclei detected in the biopsy sample, versus 2 % in the excision specimen

*ALK* status was introduced in routine practice in 2011 after it was recognized as a molecular target of crizotinib in non-small-cell lung cancer [23]. Due to lack of material, we were not able to retrospectively study all the samples obtained between 2005 and 2011 included in the study. In total, *ALK* status was evaluated in 25 patients using immunochemistry (*n* = 19), fluorescence in situ hybridization (*n* = 7) or both (*n* = 2). Discordance was observed in two former smokers. A female showed *ALK* rearrangement in the primary tumor (17 % break-apart signals) on FISH, but was negative in the metastatic brain tumor (FISH: 1 %); interestingly, *KRAS* testing led to the opposite result: detection of the G12C mutation in the metastatic but not in the primary lesion. The second case was a male, positive for *ALK* FISH in a small biopsy specimen (FISH: 25 %), whereas the *ALK* alteration was not detected in the resection specimen (FISH: 2 %): i.e., discrepancy between biopsy sample and surgical specimen of a regionally localized stage II lung cancer (Fig. 3).

### Discordance analysis

The seven cases of discordance (eight mutational discordances in a total seven patients, one patient showing discordances in both *ALK* and *KRAS* mutation status) were analyzed with respect to gender, smoking status, cancer treatment, time between the two samples from a given patient, and type of second sample (repeat primary biopsy versus biopsy of metastasis). Six discordances were found in the 26 patients sampled at two distinct sites Table 2; however, the study lacked power to demonstrate any significant difference in risk compared to the 18 patients sampled twice at the same site (6/26 versus 1/18 cases of discordance; *p* = 0.24, Fisher’s exact test). The metastases involved in these cases of discordance were located in the lung (1/6), brain (3/6), bone (1/6) or pleura (1/6). There were no other trends.

**Table 2:**
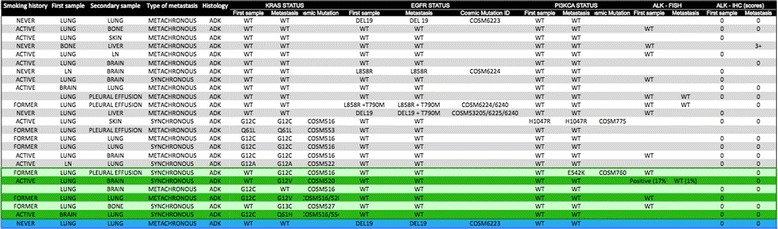
Distribution of mutations in patients with samples from different sites. Ln: lymph node; WT: wild type, patients with discordant status  one patient with two distinct synchronous lung cancers

## Discussion

Despite many recent studies of discordance in mutation status between primary lung tumors and their metastases and the feasibility of tumor rebiopsy [12], metastatic sites are rarely biopsied, as the results would currently have few if any direct therapeutic implications. Discordant responses to chemotherapy or tyrosine kinase inhibitors have also been described between primary tumors and their metastases, suggesting the existence of biological differences [24]. However, targeted therapies are usually administered on the basis of the mutational status of the primary tumor, which is most amenable to biopsy (bronchoscopy).

Major *KRAS* mutations were frequent in the present cohort (36.4 %), as generally reported in northern and western France [25]. *EGFR* activating mutations were also frequent (13.6 %): this may be explained by the fact that patients with *EGFR* activating mutations are more likely to be rebiopsied, as they progress under TKI therapy.

There was only one discordance in *EGFR* mutation, in a patient with different activating mutations at two distinct pulmonary sites considered as two synchronous primary tumors (Fig. 2). Other activating mutations were present at both sites. The p.Thr790Met (T790M) resistance mutation was found in metastases in three patients, but this was not considered as discordance because the initial activating mutation was still present in the metastasis. Our findings are slightly at odds with those of several other series which reported discordances in *EGFR* status [26–28]. Recent series also showed a very low rate of discordance in *EGFR* status, consistent with the present results [29]. The lack of discordance with respect to *EGFR* activating mutations is consistent with their “driver” status*.*

The present rate of discordance in *KRAS* mutational status (13.6 %) is consistent with that observed in several other studies [14,15,24]. We found three types of discordance: i) mutation at the primary but not the metastatic site, ii) mutation at the metastatic site, and iii) one mutation at the primary site and a different mutation in the metastasis. This variability of *KRAS* mutational status may reflect the fact that it behaves as a “passenger” mutation that, by definition, barely influences tumor outcome. We found a high rate of mutations in codons 12 and 13 of the *KRAS* gene (37.2 %), particularly the p.Gly12Cys (G12C) mutation. This mutation is usually associated with tobacco smoking [30–32]. Consistently, in the present study, at least 32 of the 44 patients had history of heavy smoking.

In one patient, the sampled metastasis harbored two different mutations: *KRAS* p.Gly12Cys (G12C) and *PI3KCA* p.Glu542Lys (E542K). This is in agreement with recent studies [29], confirming the possible coexistence of mutations, a concept that was long contested.

None of the study parameters (gender, smoking, age, anticancer treatment between samples) was significantly associated with overall risk of discordance. Although the difference could not be shown to be statistically significant, it is noteworthy that all six discordances involved patients sampled at two different sites, synchronously or metachronously, while only one involved patients sampled twice at the same site.

The discordances observed in patients sampled at two different sites might result from divergent evolution over time, with a possible influence of microenvironment and/or treatment effects. The advent of NGS (next generation sequencing) will probably extend such studies to larger populations, with dependence on the quality of the tumor tissue sampled. These new techniques are also expected to determine the molecular profile of each tumor site and to determine affiliation between two tumor sites with certainty.

Discordances between *ALK* status in primary lesions and their corresponding metastases and in multiple primary lesions have been reported in a subset of patients with NSCLC [16,17]. The present study found different types and levels of discordance in two patients unresponsive to crizotinib.

The first patient was a female who had at least a 30 pack-year history of smoking. She underwent surgical resection of brain metastasis (*ALK* negative) and, thereafter, received crizotinib for 6 months. The best response to ALK TKI administration was stable disease. ALK TKI was switched to radio-chemotherapy, which was well tolerated and led to reduction of the primary lung cancer. Although FISH is widely used as a gold standard method to diagnose *ALK*-rearranged NSCLC, it is important to remember that testing by FISH does not have 100 % sensitivity and specificity and shows cellular false-negatives and false-positives [33,34]. Here, the patient’s primary tumor tissue sample was borderline positive on FISH; the percentage of *ALK* rearrangements fell in the 15 to 20 % range. Giving the clinical history of the patient, it may be assumed that FISH led to a false-positive result.

The second patient was a male lifelong heavy smoker (35 packs-years) who developed lung cancer at 68 years of age. He was diagnosed after bronchial biopsy of a pT2N0M0 non-small-cell lung carcinoma. He underwent surgical resection of the lung cancer with standard lymphadenectomy. The disease relapsed 10 months after surgery, with lymph nodes and cerebral metastasis. At the first attempt, the patient received cisplatin-pemetrexed chemotherapy. CT scan showed remarkable lung tumor shrinkage (>50 %) and a stable cerebral lesion. After a few weeks, however, the patient asked to stop maintenance therapy, and the disease progressed rapidly. At this stage, the patient was treated with crizotinib. Despite dose reductions, the patient experienced severe renal failure that forced us to prematurely stop the targeted therapy. After 2 months of daily ALK TKI administration, the tumor manifested no response. This case is different in that the patient displayed discordance in *ALK* status between two regions of the same tumor: a small biopsy specimen showed an *ALK* rearrangement that was not detected in the surgical specimen of the corresponding regionally localized lung cancer. This illustrates the clonal evolution of lung tumors and the fact that *ALK*-positive clones sampled by biopsy may not necessarily be representative of the entire tumor. A situation exactly opposite was very recently reported by Abe and collaborators [18]. While our manuscript was under review, a paper appeared showing an intratumor heterogeneity of ALK rearrangement in a total of 7 NSCLC tumor samples (seven out of ten ALK positive tumors detected in a series of 105 mixed adenocarcinomas and 17 adenosquamous carcinomas). The authors attributed the observed differences in ALK status to the existence of different cell populations within the tumor. In contrast to the apparent stability of EGFR-activating mutations, they have evidenced a relationship between ALK status and certain histologic subtypes. As stated by the authors, this could suggest that rearrangement of the ALK gene is a late event of tumorigenesis [35]. Together, these observations suggest a limitation of single biopsy-based analyses for predictive biomarker tracking and personalized medicine.

## Conclusions

In conclusion, this study confirms the substantial rate of discordance in mutational status between primary tumors and their metastases in patients with non-small-cell lung cancer. Discordance mainly concerned *KRAS*, an oncogene frequently mutated in lung cancer, particularly in smokers. *KRAS*-positive lung cancer patients are among the most refractory to available treatments, but efforts to develop new therapies for these patients, including anti-MEK drugs, are particularly intensive. The present findings indicate that, with the development of successful targeted therapies, *KRAS*-positive patients would benefit from genetic testing of different samples. Currently, and by contrast, the stability of *EGFR* status between primary sites and metastases confirms that there is no reason to systematically rebiopsy all patients, as the results would have no direct therapeutic implications other than in clinical trials. The discordance in *ALK* rearrangement found between a small biopsy and the corresponding surgical specimen shows that it is important to accumulate information about the biological behavior of infrequent genetic alterations with predictive value. Intra-tumor heterogeneity is another major source of concern in therapeutic resistance.

## Additional file

Additional file 1: SNaPshot Multiplex analysis of KRAS codons 12 and 13 (HUGO Gene Nomenclature Committee #6407). (ODP 128 kb)

## Abbreviations

ALK: anaplastic lymphoma kinase
BRAF: V-RAF murine sarcoma viral oncogene homolog
CT scan: computed tomography scan
EGFR: epidermal growth factor receptor
FFPE: formaldehyde fixed and paraffin embedded
FISH: fluorescence in situ hybridization
HER2: V-ERB-B2 avian erythroblastic leukemia viral oncogene homolog 2
KRAS: V-KI-RAS2 kirsten rat sarcoma viral oncogene homolog
NSCLC: non-small-cell lung cancer
PIK3CA: phosphatidylinositol 3-kinase, catalytic alpha
TKI: tyrosine kinase inhibitor
